# Virulence Structure and Genetic Diversity of *Blumeria graminis* f. sp. *avenae* from Different Regions of Europe

**DOI:** 10.3390/plants11101358

**Published:** 2022-05-20

**Authors:** Magdalena Cieplak, Aleksandra Nucia, Tomasz Ociepa, Sylwia Okoń

**Affiliations:** Institute of Plant Genetics, Breeding and Biotechnology, University of Life Science, 20-950 Lublin, Poland; magdalena.cieplak@up.lublin.pl (M.C.); aleksandra.nucia@up.lublin.pl (A.N.); tomasz.ociepa@up.lublin.pl (T.O.)

**Keywords:** genetic diversity, molecular markers, oat, pathogenicity, powdery mildew

## Abstract

The structure and dynamics of changes in pathogen populations can be analysed by assessing the level of virulence and genetic diversity. The aim of the present study was to determine the diversity of *Blumeria graminis* f. sp. *avenae* populations. Diversity and virulence of *B. graminis* f. sp. *avenae* was assessed based on 80 single-spore isolates collected in different European countries such as Poland (40 isolates), Germany (10), Finland (10), Czech Republic (10) and Ireland (10) using ISSR (*Inter-Simple Sequence Repeats*) and SCoT (*Start Codon Targeted*) markers. This work demonstrated differences in virulence of *B. graminis* f. sp. *avenae* isolates sampled from different countries. Molecular analysis showed that both systems were useful for assessing genetic diversity, but ISSR markers were superior and generated more polymorphic products, as well as higher PIC and RP values. UPMGA and PCoA divided the isolates into groups corresponding with their geographical origin. In conclusion, the low level of genetic differentiation of the analysed isolates has suggested that the evolution of *B. graminis* f. sp. *Avenae* population is slow, and thus the evolutionary potential of the pathogen is low. This work paves the way for future studies on *B. graminis* f. sp. *Avenae* population structure and dynamics based on genetic variability.

## 1. Introduction

Oat (*Avena sativa* L.) is a species susceptible to many abiotic and biotic factors, among which fungal pathogens play a particular role. One of them is oat powdery mildew caused by *Blumeria graminis* f. sp. *Avenae*, an obligate biotrophic pathogen [1,2,3]. The destructive foliar disease of oat has a significant negative impact on yield and its quality, resulting in poor feed grade. Yield losses caused by powdery mildew can reach up to 40%, however, on average they amount to 5–10% [4,5,6]. Therefore, developing resistant cultivars can be an effective crop protection strategy without the use of fungicides. Nevertheless, pathogen–host interactions are highly complicated and dynamic processes; therefore, knowledge of virulence frequencies, population structure and genetic diversity of pathogens is necessary to achieve this goal.

Oat powdery mildew reproduces asexually for most of its life and spreads primarily by conidia. Sexual reproduction, which occurs once a year, enables this species to survive. During autumn when temperatures drop, the pathogen produces ascospores, which are released in the summer when it is warm. Ascospores can survive winter on plant residues and may be a source of infection in the spring [7]. The *B. graminis* f. sp. *avenae* population is constantly evolving towards greater virulence complexity through mutation, migration and recombination. The annual sexual reproduction cycle leads to the formation of new allelic combinations, and subsequent cycles of asexual reproduction may result in an increase in the frequency of alleles determining greater virulence of the pathogen. In addition, climate change and associated extreme weather conditions make it easier for *B. graminis* spores to spread over long distances. These phenomena contribute to the formation of new and aggressive pathotypes [8,9].

Cultivation of plants with genetic resistance is a sustainable method of controlling powdery mildew. Therefore, an important aspect of breeding programmes is the characterisation of plant pathogen populations by analysing the dynamics of genetic evolution, studying the spread of pathogens between different regions and determining the variability of pathogenic isolates. In this manner, the effectiveness of resistance genes can be continuously monitored, preventing cultivars from losing resistance as early as possible [10]. Detailed knowledge of the genetic diversity of the pathogen present in a given area allows the determination of its evolutionary potential. A pathogen population with high evolutionary capacity is more likely to overcome genetic resistance than a population with low evolutionary potential. The latter can be analysed by assessing the level of virulence, and on the basis of genetic variation, by determining the diversity within and between populations [10,11].

Molecular markers play one of the key roles in understanding the virulence frequency of *B. graminis*. In particular, systems based on DNA molecular markers allow the analysis of genetic diversity of pathogens. Such markers have been shown to be useful in analysing the genetic diversity of different plant pathogens [10,12,13,14]. Various molecular markers have so far been used to assess the genetic structure of *B. graminis* f. sp. *Hordei* and *B. graminis* f. sp. *Tritici* populations These studies were based on the SSR (*Simple Sequence Repeat*) and ISSR (*Inter-Simple Sequence Repeats*) systems, and SRAP (*Sequence-Related Amplified Polymorphism*) and SNP (*Single Nucleotide Polymorphism*) markers [10,15,16,17].

Studies on the virulence of oat powdery mildew have already been reported several times in the literature [18,19,20]. However, there is no information on research into the genetic diversity of *B. graminis* f. sp. *avenae*. Hence, the objective of the present work was to determine the diversity of *B. graminis* f. sp. *avenae* populations from different regions of Europe. The diversity of *B. graminis* f. sp. *avenae* was assessed using ISSR and SCoT markers and based on the virulence of the pathogen collected in different European countries

## 2. Results

### 2.1. Virulence Analysis

*B. graminis* f. sp. *avenae* isolates collected in Poland, Germany, Czech Republic, Finland and Ireland showed varying levels of virulence compared to the control forms with described resistance genes and effective sources of resistance (Table 1). All analysed isolates showed the highest average value of virulence frequency against the *Pm1*, *Pm6* and *Pm11* genes. In contrast, all analysed isolates were avirulent or showed low virulence towards the *Pm2*, *Pm4*, *Pm5* and *Pm7* genes and resistance sources identified in *A. sterilis* CN67383 and *A. strigosa* Pl51586 genotypes.

Isolates from different countries differed in the frequency of virulence. The population from the Czech Republic was virulent to the *Pm6* and *Pm11* genes and to the cultivar Canyon carrying the *Pm7* gene. It showed a moderate virulence frequency against *Pm1* and *Pm3*. The isolates collected from Finland were virulent to the *Pm1*, *Pm6*, *Pm9*, *Pm10*, *Pm11* genes and the *Pm7* gene from the cultivar Canyon. They showed a moderate virulence frequency towards *Pm3* and the combination of *Pm3* and *Pm8* resistance genes. The population from Ireland broke the resistance conferred by the *Pm1*, *Pm10* and *Pm11* genes. It exhibited a moderate level of virulence against *Pm6* and *Pm9* and was avirulent to the remaining genes. The population from Germany was completely virulent to the *Pm1*, *Pm3*, *Pm6* and *Pm3 + 8* genes. These isolates presented a moderate virulence against the *Pm5*, *Pm9*, *Pm10* and *Pm11* genes and the cultivar Canyon carrying the *Pm7* gene. The Polish population characterised the greatest diversity of virulence frequency against resistance genes. Isolates from Poland displayed high levels of virulence to *Pm1*, *Pm3*, *Pm6* and *Pm3 + 8* and a moderate virulence against *Pm9*, *Pm10*, *Pm11*, *A. sterilis* and the cultivar Canyon carrying *Pm7*. Several isolates were virulent to the *Pm5* and *Pm7* genes and to *A. strigosa*. None of the analysed isolates broke the resistance conditioned by *Pm4*.

The analysed *B. graminis* f. sp. *avenae* isolates were classified into 37 different pathotypes based on their virulence. The most abundant was the TBBB pathotype, with a frequency of 0.075. It was represented by isolates from Poland and Germany (Supplementary Material Table S1). The highest number of pathotypes was identified in the Polish population (22). The populations from Ireland, Finland and the Czech Republic were represented by three pathotypes each, whereas the population from Germany was represented by five pathotypes. Among thirty-seven pathotypes, one common pathotype was identified for the populations from Poland and the Czech Republic, one for the populations from Poland and Finland, and two for the populations from Poland and Germany.

Different types of diversity parameters were calculated for individual populations based on their virulence. All the results obtained are presented in Table 2. They clearly indicated that the population of *B. graminis* f. sp. *avenae* in Poland was the most diverse. This could be due to the large number of *B. graminis* f. sp. *avenae* isolates representing this population. Among the remaining populations, isolates collected from Germany showed the greatest diversity.

Nei’s distance (N) and Nei’s standardized coefficient of gene differentiation (Nei’s Gst) showed that the populations from Ireland and Germany were the most distant from each other. The smallest genetic distance was observed between the populations from Poland and Germany. The gene flow (Nm) value for these populations was the highest and amounted to 7.83, indicating a high migration of alleles between these regions. The lowest gene flow was observed between the populations from Ireland and Germany (1.42), which could be related to the geographic barrier between these areas (Table 3).

### 2.2. Genetic Diversity Based on ISSR and SCoT Markers

Of the 30 examined ISSR primers, 12 detected polymorphisms between individuals. A total of 175 clearly distinguishable amplification products were found. Of these, 131 were polymorphic, with an average of 10.91 bands for each primer. The percentage of polymorphic bands for all primers was higher than 50%. The resolving power of the primers ranged from 15.46 to 4.1; the PIC scores ranged from 0.37 to 0.22 (Table 4).

A total of 236 amplification fragments were obtained using 14 of the 30 tested SCoT primers. Of these, 122 were polymorphic, with an average of 8.71 bands for each primer. The percentage of polymorphic bands for all primers was higher than 50%. The resolving power of the primers ranged from 13.45 to 4.67; the PIC score ranged from 0.36 to 0.13 (Table 4).

The level of genetic diversity of *B. graminis* f. sp. *avenae* isolates from five different countries was determined using ISSR and SCoT markers. The analysed isolates were considered as five distinct populations according to their geographic origin. Both marker systems indicated that the population from Poland was the most diverse. The highest number of polymorphic bands and population-specific amplification products was identified in this population. Both ISSR and SCoT analyses showed that the population from Finland was the least diverse. The lowest level of band polymorphism was observed for this population and the lowest level of diversity was confirmed by the Shannon index and expected heterozygosity (Table 5).

The analysis of molecular variance (AMOVA) of ISSR and SCoT polymorphisms revealed that the genetic divergence was mainly derived from within the population (97% ISSR, 87% SCoT).

**Figure 1:**
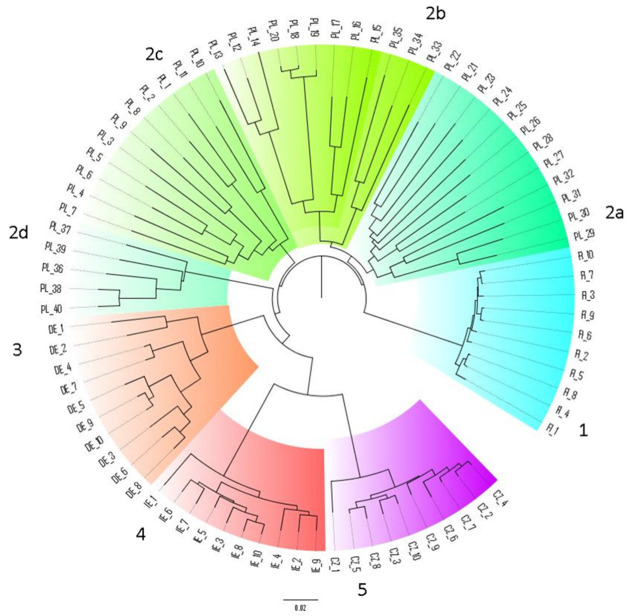
UPGMA analysis of 80 *B. graminis* f. sp. *avenae* isolates. Cluster 1—isolates from Finland. Cluster 2—isolates from Poland: 2a—central part of the country; 2b—southern part of the country; 2c—eastern part of the country; 2d—western part of the country. Cluster 3—isolates from Germany. Cluster 4—isolates from Ireland. Cluster 5—isolates from Czech Republic.

The polymorphisms of ISSR and SCoT markers were used to calculate the genetic distance between *B. graminis* f. sp. *avenae* populations. The analysis based on ISSR markers showed that the populations form Poland and Germany were characterised by the lowest genetic distance, as well as the highest level of gene flow. The highest genetic distance was determined for the populations form Ireland and Finland. The analysis of genetic distance based on SCoT markers also indicated that the Polish and German populations were the closest to each other, while the highest distance was calculated between populations from Ireland and Germany (Table 3, Figure 1).

Cluster analysis based on both ISSR and SCoT markers demonstrated a tendency for *B. graminis* f. sp. *avenae* isolates to group according to their geographic origin. Independent dendrograms based on ISSR and SCoT analysis (data not shown) and a combined dendrogram based on both marker systems (Figure 1) showed similar results. The isolates clustered into five major groups, representing five different populations. The length of the dendrogram branches reflected a genetic similarity level within the populations and clearly confirmed that the population from Finland was the least diverse, while the Polish population was characterised by the highest diversity. Moreover, it was observed that the Polish population clustered into subgroups depending on the region from which the pathogen samples were collected. The Polish subpopulations were also more differentiated than the populations from the Czech Republic, Ireland or Finland.

**Figure 2:**
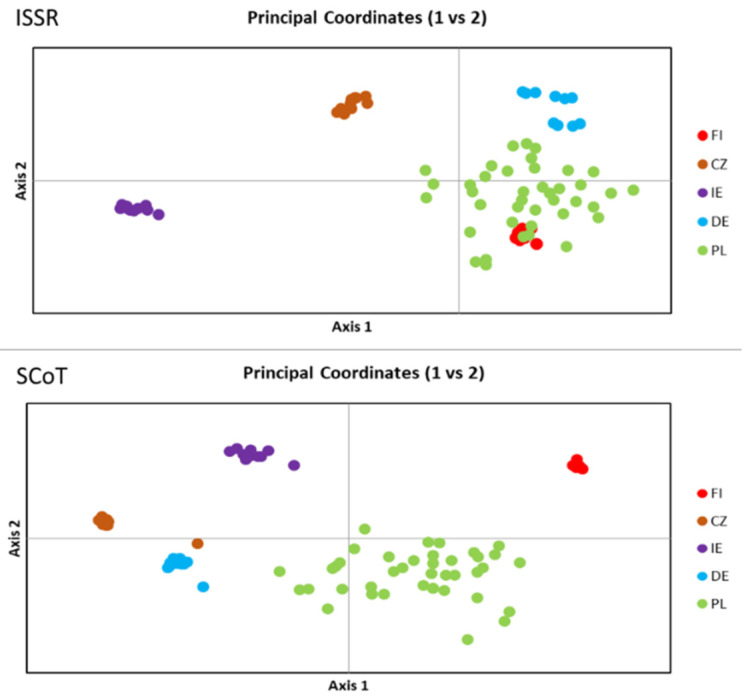
The result of PCoA analysis preformed based on genetic distance among *B. graminis* f. sp. *avenae* isolates.

PCoA analysis was performed based on the genetic distance obtained from ISSR and SCoT data separately (Figure 2). Both methods tended to group the isolates according to their geographic origin. However, clustering showed some differences that reflected genetic distances measured using two different methods. The genetic distance calculated on the basis of ISSR polymorphisms clearly demonstrated the distinctiveness of isolates from Ireland, the Czech Republic and Germany. The isolates from Finland grouped together with the isolates from Poland, forming a very small cluster. On the other hand, the genetic distance calculated on the basis of SCoT markers clearly divided the analysed isolates into groups corresponding to their geographical origin. In both cases, it was clearly visible that the population from Poland was the most diverse.

## 3. Discussion

Knowledge of the genetic structure of pathogen populations provides insight into the evolutionary process that shaped the population in the past. On the other hand, this knowledge also allows us to evaluate the future evolutionary potential of pathogen populations. Therefore, characterisation of pathogen populations should be carried out on a regular basis, especially in those cases where pathogens cause large losses in yield quality and quantity. Knowledge of evolutionary potential may prove significant in developing pathogen control strategies, both based on the use of resistance genes and selection of fungicides and their doses [11].

The structure and dynamics of changes in pathogen populations can be analysed using two different approaches. The first is based on the assessment of the virulence level of the pathogen population [19,21,22,23,24,25,26]. This approach allows us to determine the effectiveness of resistance genes used in breeding and to estimate the possibility of breaking this resistance by emerging pathogen races. This study revealed large differences in the levels of virulence of *B. graminis* f. sp. *Avenae* isolates collected from different parts of Europe. These differences suggested that the effectiveness of resistance genes varied from region to region, e.g., for many years, the *Pm3* gene was considered ineffective in Poland [18,19,20] but in the current study its high resistance against isolates from Ireland and Finland was demonstrated. This research also showed that the virulence level of the population collected in Poland was very similar to that identified in previous years [18,19], indicating that changes in the pathogen population were slow. The population from Poland showed the highest similarity to the population collected in Germany, thus it could be assumed that these populations were similar in terms of the rate changes. The highest level of gene flow and the highest number of shared pathotypes were observed between these two populations.

The second approach in pathogen population analysis is the assessment of genetic diversity based on molecular markers [27,28,29]. This study is the first attempt to investigate the genetic diversity of *B. graminis* f. sp. *avenae* populations from different regions of Europe. Literature data show that so far the genetic structure of *B. graminis* populations infecting various cereal species has been studied using gene sequence analysis or molecular markers such as SSR, ISSR, RAPD or SRAP [10,15,30,31,32]. The B. *graminis* genome is rich in numerous repetitive sequences [33,34]; therefore, ISSR markers [35], which amplify regions between repetitive sequences, have been selected in the present study. These markers have been successfully utilised to analyse the genetic diversity of various pathogen populations [36,37,38]. The first step of our research was the selection of markers amplifying repetitive and polymorphic products. Screening tests showed that the best amplification was initiated by primers composed of GA and CA motifs, while primers with AT and CT dinucleotides did not amplify products on the *B. graminis* f. sp. *avenae* DNA template (data not shown). It can be speculated that the repetitive regions in the *B. graminis* f. sp. *avenae* genome are mainly composed of GA and CA motifs. SCoT markers were the second system selected to analyse the genetic variability in the *B. graminis* f. sp. *avenae* population. Start codon targeted (SCoT) polymorphism is a simple and novel DNA marker technique described by Collard and Mackill [39]. SCoT markers amplify gene-targeted fragments which allows us to obtain new information corresponding to biological data, while random DNA markers such as RAPD, AFLP or ISSR do not provide such data. Different pathogens were studied using SCoT markers [40,41].

Both marker systems were useful for analysing the genetic structure of *B. graminis* f. sp. *avenae* populations. However, ISSR markers amplified more polymorphic products, and were characterised by higher PIC and RP values than SCoT markers. These results indicate that ISSR markers are a better tool for studying the genetic diversity of *B. graminis* populations which is consistent with Liu et al. [10]. The latter authors obtained higher PIC and RP values, as well as a percentage of polymorphic products for ISSR markers compared to SRAP markers. Studies conducted on various pathogens have proven that SRAP markers are a good tool to identify polymorphism and genetic variation [42,43,44]. SRAP markers were initially also selected together with ISSR markers in the current study. Nevertheless, after preliminary screening, no satisfactory polymorphisms were detected between *B. graminis* f. sp. *avenae* isolates; therefore, SCoT markers were chosen for further analyses (data not shown).

The analysed *B. graminis* f. sp. *avenae* isolates were divided into five distinct populations depending on their place of origin. Both marker systems indicated that the population from Poland was the most diverse. However, this could be due to the large number of isolates representing this population. We observed a high variation between the Polish isolates, which was likely due to the different geographic regions from which they originated. Among the remaining populations, isolates from Germany were the most diverse. The population from Finland, for which the lowest diversity values were identified based on both ISSR and SCoT markers, was the most homogeneous.

PCoA analysis based on both ISSR and SCoT markers divided *B. graminis* f. sp. *avenae* isolates into groups according to their geographic origin. Clustering correlated with geographic origin of *B. graminis* f. sp. *tritici* isolates was also observed by Liu et al. [10], who utilised both ISSR and SRAP markers. These authors, as in our study, did not observe clustering associated with virulence. This indicated that molecular markers such as ISSR, SRAP [10], SNP [16] and SCoT, used in this study, identified variation not associated with pathogenicity. Wu et al. [31] used EST-SSR markers to characterise the *B. graminis* f. sp. *tritici* population and showed that polymorphisms detected using EST-SSR markers were correlated with the pathogen virulence in 58% of the analysed isolates. However, this correlation was not observed for the remaining isolates. The authors suggested that the level of pathogen virulence depended on many factors and that the gene sequence alone could not determine pathogenicity. The complex interaction between the plant and pathogen and the environmental impact may result in the weak correlation of polymorphisms generated by molecular markers with pathogen virulence.

Grouping associated with geographic regions indicated the genetic distinctiveness of the analysed populations. This could be observed for populations separated by geographic distance and natural barriers (Ireland and Finland). The latter probably also influenced the highest similarity found between populations in Poland and Germany and not in the Czech Republic. The genetic distinctiveness of the analysed populations was also confirmed by AMOVA. This analysis showed that the variability identified among the *B. Graminis* f. sp. *Avenae* isolates tested was largely based on the intra-population variation. This was also indicated by the low level of gene flow between the analysed populations. Similar results were obtained for *B. graminis* f. sp. *Tritici* [10] and *B. graminis* f. sp. *Hordei* [15] populations, for which most of molecular variation also occurred between individuals within individual populations.

## 4. Materials and Methods

Powdery mildew-symptomatic oat leaves were collected in 2020 from five European countries, including Poland, the Czech Republic, Germany, Ireland and Finland. The sampling sites differed from one another in terms of climate and geography. These places reflected the different conditions of Europe in which the powdery mildew of oats is present. The isolates were prepared under laboratory conditions according to the previous methodology described by Hsam et al. [45,46]. In total, 80 single spore isolates were used in the experiment. Samples collected from different countries were treated as a separate population. Populations from Czech Republic, Germany, Ireland and Finland were each represented by 10 isolates. Population from Poland was represented by 40 isolates collected in different parts of the country.

### 4.1. Virulence Analysis

To analyse the virulence of the pathogen populations, host–pathogen tests were carried out using eleven oat genotypes, with known powdery mildew resistance genes and two genotypes with effective sources of resistance identified in our previous study [47,48]. The cultivar Fuchs, without any powdery mildew resistance genes, were used as a susceptible control. All control genotypes were seeded into plug trays filled with universal substrate and germinated. After ten days, the leaf fragments of the analysed genotypes were placed on 12-well culture plates with benzimidazole agar (6 g of agar per 1 L of water and 35 mg/L of benzimidazole). Plates with leaf segments were inoculated in an inoculation tower by placing about 500–700 of *B. graminis* f. sp. *avenae* spores per 1 cm^2^. Then, the dishes were incubated in a growing chamber at about 17 °C and illuminance of approximately 4 kLx.

Reaction type on each differential was determined 10 days after inoculation and scored according to a 0–4 modified scale [49]: where 0 = no infection, no visible symptoms; 1 = highly resistant, fungal development limited, no sporulation; 2 = moderately resistant, moderate mycelium with some sporulation; 3 = moderately susceptible, extensive mycelium, more sporulation; and 4 = highly susceptible, large colonies and abundant sporulation. If disease symptoms were scored as 0, 1 or 2, the isolates were classified as avirulent to known genes against oat powdery mildew. If disease symptoms were scored as 3 or 4, the isolates were classified as virulent.

Parameters for comparing all *B. graminis* f. sp. *avenae* isolates were calculated on the basis of isolate virulence patterns on the set of differential genotypes. Virulence frequency (*p*) as *p = x/n* (where *x* is the number of times a virulent reaction type was detected and *n* is the total number of samples tested) was calculated for each isolate.

Diversity within the populations was assessed using different types of parameters: genetic diversity was measured by Simpson (Si) and Shannon (Sh) based on the pathotype structure of the populations; gene diversity was measured by the Nei index (Hs) which is equivalent to a measurement of the average dissimilarity within a population (ADW_m_) regarding the simple mismatch coefficient m, and the Nei gene distance (N) based on the population virulence; and genetic diversity (KW_m_) and distance (KB_m_) measured by the Kosman indices, based on the population pathotype and virulence structure [50,51,52]. Pairwise distance between populations was assessed using Nei distance (N) and Nei’s Gst. All computations of populations parameters were performed with the VAT software [52,53]. The gene flow index (Nm) was estimated based on the Gst values according to the formula: Nm = 0.5(1 − Gst)/Gst.

The virulent and avirulent types observed were transformed into binary coding matrix for computational analysis. Based on binary matrix, the analysed *B. graminis* f. sp. *avenae* isolates were classified into appropriate pathotypes according to the methodology described by Okoń et al. [20].

### 4.2. Genetic Diversity Based on ISSR and SCoT Molecular Markers

Genomic DNA from *B. graminis* f. sp. *avenae* spores were isolated according to the methodology described by Feehan et al. [54].

Inter-microsatellite sequence analysis (ISSR) was performed using 30 primers [35]. A 10 µL volume of the reaction mixture consisted of water, 1× concentrated reaction buffer, 2.5 mM magnesium chloride, 0.2 mM dNTP, 0.5 mM primer, 0.46 U Taq polymerase and 40 ng of genomic DNA. Amplification was carried out in a Biometra T1 thermal cycler programmed for 3 min at 94 °C of initial denaturation, 35 cycles: 94 °C—30 s, 45 s in the first three cycles at 54 °C; 53 °C in three successive ones; 52 °C in others; and 72 °C for 2 min, with a final extension at 72 °C for 5 min.

The analysis of genetic similarity was based on SCoT marker systems [55]. A total of 30 primers were used for screening tests. Reaction mixtures contained 1× PCR Buffer (10 mM Tris pH 8.8, 50 mM KCl, 0.08% Nonidet P40) (Thermo Scientific, Walthman, MA, USA), 160 μM of each dNTP, 800 pM oligonucleotide primer, 1.5 mM MgCl_2_, 60 ng of template DNA and 0.5 U Taq DNA Polymerase (Thermo Scientific), in a final reaction mixture of 10 μL. Amplification was carried out in a Biometra T1 thermal cycler programmed for 3 min at 94 °C of initial denaturation, 35 cycles: 94 °C—1 min; 50 °C—1 min; and 72 °C—2 min, with a final extension at 72 °C for 5 min.

Amplification products were separated by electrophoresis on 1.5% agarose gels containing 0.1% EtBr (1.5 h, 120 V). Fragments were visualised under a UV transilluminator and photographed using the PolyDoc System. GeneRuler^TM^ 100 bp DNA Ladder Plus was used to establish the molecular weights of the products.

PCR-amplified ISSR and SCoT products were scored as present (1) or absent (0) from the photographs. Only clear and reproducible products were scored. The level of polymorphism of the primer (polymorphic products/total products) and relative frequency of polymorphic products (genotypes where polymorphic products were present/total number of genotypes) [56] were calculated. Resolving power of the primer was calculated using the formula: Resolving power (Rp) = Σ Ib (band informativeness). Band informativeness was calculated for each band scored by the primer individually. Ib = 1 − [2(0.5 − p)], p is the proportion of occurrence of bands in the genotypes out of the total number of genotypes [57]. Polymorphic information content (PIC) was calculated by applying the simplified formula [58]: PIC = 2fi(1 − fi), where fi is the percentage of the amplified band present. The percent of polymorphic loci (P %), frequency of each allele and the average number of alleles at the locus (N_a_) were established. An effective number of alleles at the locus (N_e_) [59] and expected heterozygosity (H_e_) [60] were estimated. Based on Shannon’s index (I) [61], the level of intra-population differentiation was determined. The genetic distance between the examined individuals was calculated [62] and a PCoA was made. Additionally, a molecular analysis of variance (AMOVA) was performed. The mentioned parameters of genetic variability were calculated using GeneAlex ver. 6.0 [63]. Gene flow (Nm) and Nei genetic identity and Nei genetic distance between analysed populations were estimated using PopGene 32 [64].

Dendrogram representing genetic diversity was constructed by performing unweighted pair-group analysis with arithmetic averages (UPGMA) using PAST 3.16 [65].

## 5. Conclusions

Two approaches were employed in this work to analyse the diversity of the pathogen population. To our knowledge, this is the first study that has been conducted on the *B. graminis* f. sp. *avenae* population. Our research has revealed that the level of resistance to powdery mildew in oat differs depending on the region. Therefore, combining resistance genes into pyramids in oat breeding can lead to long-term resistance under different environmental conditions. Furthermore, virulence analysis showed that the *B. graminis* f. sp. *avenae* population from Poland had a similar level of virulence as the populations studied in previous years. This suggests that the pathogen population is changing slowly.

Polymorphisms of ISSRs and SCoT markers grouped *B. graminis* f. sp. *avenae* isolates according to their geographic origin. Consequently, it can be concluded that the level of genetic differentiation of the pathogen does not depend on the level of virulence, but on its origin. The low level of genetic differentiation of the analysed isolates suggests that the evolution of the *B. graminis* f. sp. *avenae* population is slow, and thus the evolutionary potential of the pathogen is low, which may result from the lack of environmental pressure on the pathogen. Oat cultivars are characterised by a low level of resistance to powdery mildew [46,66]; therefore, the pathogen does not encounter barriers in the form of effective and strong resistance genes. If no new cultivars with effective resistance genes appear in oat breeding in the near future, extensive changes in the genetic makeup of this pathogen population shall not be expected, which will be translated into small and slow changes in the level of virulence.

## Figures and Tables

**Table 1:** Virulence frequencies of *Blumeria graminis* f. sp. *avenae* populations.

Differential	Gene	Frequency (%)					
		Czech Republic CZ	Finland FI	Ireland IE	Germany DE	Poland PL	Average
Jumbo	*Pm1*	33	100	100	100	93	85
CC3678	*Pm2*	0	0	0	0	0	0
Mostyn	*Pm3*	56	40	0	100	80	55
Av1860	*Pm4*	0	0	0	0	0	0
Am27	*Pm5*	0	0	0	50	3	11
Bruno	*Pm6*	100	100	50	100	83	87
APR122	*Pm7*	0	0	0	0	8	2
Canyon	*Pm7*	0	50	0	100	83	47
Rollo	*Pm3 + Pm8*	0	100	40	20	18	36
AVE2406	*Pm9*	0	100	100	20	28	50
AVE2925	*Pm10*	100	100	100	40	50	78
CN113536	*Pm11*	0	0	0	0	25	5
CN67383	UA.ster.	100	100	0	20	30	50
Pl51586	UA.stri.	0	0	0	0	10	2
Fuchs	-	100	100	100	100	100	100

**Table 2:** Diversity parameters of the analysed *B. graminis* f. sp. *avenae* populations.

Population	Number of Isolates	No. of Different Pathotypes	Hs	Si	Sh	KW_m_
PL	40	22	0.23	0.95	0.86	0.31
CZ	10	3	0.06	0.57	0.43	0.10
FI	10	3	0.07	0.58	0.41	0.12
IE	10	3	0.07	0.58	0.41	0.12
DE	10	5	0.13	0.74	0.64	0.20

Hs—gene diversity; Si—Simpson index; Sh—Shannon normalized index; KW_m_—Kosman index.

**Table 3:** Nei’s (N) distance, Nei’s standardized coefficient of gene differentiation (Nei Gst) and gene flow (Nm) between analysed *B. graminis* f. sp. *avenae* populations.

	Virulence			Molecular Markers			
Populations	N	Nei Gst	Nm	Distance Based on ISSR	Distance Based on ISSR	Nm (ISSR)	Nm (SCoT)
FI-CZ	0.22	0.19	2.12	0.233	0.261	0.110	0.830
FI-DE	0.26	0.24	1.63	0.279	0.264	0.099	0.154
FI-IE	0.16	0.16	2.71	0.349	0.199	0.099	0.121
FI-PL	0.20	0.19	2.17	0.185	0.178	1.277	0.998
IE-CZ	0.26	0.23	1.66	0.264	0.125	0.141	0.242
IE-DE	0.31	0.26	1.42	0.338	0.181	0.237	0.289
IE-PL	0.21	0.19	2.08	0.231	0.143	0.849	1.055
DE-CZ	0.25	0.23	1.68	0.221	0.137	0.330	0.352
DE-PL	0.03	0.06	7.83	0.127	0.123	1.851	1.232
PL-CZ	0.17	0.17	2.37	0.168	0.144	0.999	1.002

**Table 4:** Characteristics of ISSR and SCoT primers used to assess the genetic diversity of the analysed *B. graminis* f. sp. *avenae* isolates.

Assay	Primer	Primer Sequence	Amplified Bands	Polymorphic Bands	Percentage of Polymorphic Bands (%)	PIC	RP
ISSR	SR39	GAGAGAGAGAGAGAGAGG	12	11	91.67	0.37	11.27
	SR41	AGAGAGAGAGAGAGAGAGAGC	14	10	71.43	0.24	8.7
	SR60	CACCACCACCACCACCACCACT	13	13	100	0.33	11.2
	SR17	GAGAGAGAGAGAGAGAYC	21	15	71.43	0.27	15.45
	SR22	CACACACACACACACAG	14	10	71.43	0.27	10.32
	SR31	AGAGAGAGAGAGAGAGAGYC	16	12	75.00	0.27	12.52
	SR37	ACACACACACACACACC	18	15	83.33	0.28	12.82
	SR40	ACACACACACACAC ACT	11	6	54.55	0.24	5.02
	SR42	AGAGAGAGAGAGAGAGYA	13	9	69.23	0.30	8.3
	SR46	GAGAGAGAGAGAGAGAGAGAA	16	12	75.00	0.31	11.52
	SR61	ACACACACACACACACACG	11	6	54.55	0.22	4.1
	SR86	CACACACACACACA CAT	16	12	75.00	0.24	14.9
	Average		14.58	10.92	74.39	0.28	10.51
SCoT	SCoT12	ACGACATGGCGACCAACG	24	14	60.87	0.18	13.45
	SCoT13	ACGACATGGCGACCATCG	17	10	58.82	0.21	11.4
	SCoT14	ACGACATGGCGACCACGC	12	6	50.00	0.15	7.87
	SCoT18	ACCATGGCTACCACCGCC	16	8	50.00	0.21	6.67
	SCoT19	ACCATGGCTACCACCGGC	16	8	50.00	0.19	6.75
	SCoT21	ACGACATGGCGACCCACA	17	1	41.18	0.12	6.35
	SCoT22	AACCATGGCTACCACCAC	12	10	83.33	0.36	10.47
	SCoT23	CACCATGGCTACCACCAG	16	8	50.00	0.17	4.67
	SCoT26	ACCATGGCTACCACCGTC	19	12	63.16	0.25	12.87
	SCoT32	CCATGGCTACCACCGCAC	14	7	50.00	0.18	8.02
	SCoT33	CCATGGCTACCACCGCAG	15	10	66.67	0.26	10.3
	SCoT34	ACCATGGCTACCACCGCA	15	7	46.67	0.18	5.92
	SCoT83	CAATGGCTACCACTAACG	20	8	40.00	0.13	6.50
	SCoT90	CCATGGCTACCACCGGCA	23	13	56.52	0.23	12.75
	Average		16.86	8.71	54.80	0.20	8.86

**Table 5:** The genetic variability parameters of *B. graminis* f. sp. *avenae* populations based on ISSR and SCoT markers. P %—percent of polymorphic loci; N_a_—number of alleles at the locus; N_e_—number of effective alleles at the locus; H_e_—expected heterozygosity; I—Shannon’s index.

Population	(P %)	(N_a_)	(N_e_)	(H_e_)	(I)
ISSR					
FI	4.60%	0.724	1.023	0.014	0.021
CZ	5.75%	0.638	1.049	0.026	0.037
IE	9.20%	0.690	1.064	0.036	0.053
DE	25.29%	1.000	1.135	0.081	0.123
PL	69.54%	1.684	1.458	0.262	0.386
SCoT					
FI	2.55%	0.821	1.014	0.008	0.012
CZ	8.94%	0.851	1.054	0.031	0.046
IE	8.51%	0.847	1.059	0.033	0.048
DE	15.74%	0.953	1.082	0.048	0.074
PL	53.62%	1.536	1.306	0.181	0.272

## Data Availability

Data are available upon request from the corresponding authors.

## Supplementary Materials

The following supporting information can be downloaded at: https://www.mdpi.com/article/10.3390/plants11101358/s1. Table S1: Pathotypes identified among analysed *B. graminis* f. sp. *avenae* isolates.
