# Supplementary material for: Virulence Structure and Genetic Diversity of Blumeria graminis f. sp. avenae from Different Regions of Europe
Source: Plants (Basel). 2022 May 20;11(10):1358. doi: 10.3390/plants11101358 (PMC9145444; doi:10.3390/plants11101358)
Supplement: Supplementary file 1 [file plants-11-01358-s001.zip › plants-1716547-supplementary.pdf]

Supplementary material Table S1. Pathotypes identified among analysed *B. graminis* f.sp. *avenae* isolates.

	pathotype	frequency	populations				
			CZ	FI	IE	DE	PL
1	DBMB	0,025	2				
2	FBBB	0,0125					1
3	HBDB	0,0125					1
4	JBMB	0,0625	5				
5	KBBB	0,0125					1
6	LBFB	0,0625			5		
7	NBBQ	0,025					2
8	NBCG	0,0125					1
9	NBCL	0,0125					1
10	NBDL	0,0125					1
11	NBFB	0,0125			1		
12	NBKB	0,05			4		
13	NBMB	0,05	3				1
14	NBRB	0,0125					1
15	NBTB	0,0625		5			
16	PBTB	0,0125		1			
17	RBBB	0,025					2
18	RBDB	0,025					2
19	RBFB	0,0125					1
20	RBTB	0,0125					1
21	TBBB	0,075				2	4
22	TBBL	0,0375					3
23	TBCB	0,025					2
24	TBCG	0,0125					1
25	TBHB	0,025				2	
26	TBKB	0,025					2
27	TBKL	0,0125					1
28	TBLL	0,0125					1
29	TBMB	0,05					4
30	TBPB	0,025				1	1
31	TBTB	0,0625		4			1
32	TCBB	0,0125					1
33	TCCB	0,0125					1
34	TCLL	0,0125					1
35	TDBB	0,05				4	
36	TDPB	0,0125				1	
37	TDRB	0,0125					1
